# Knowledge Gaps in the Definition of Threats for the Red List Assessment of European Freshwater-Dependent Fish Species

**DOI:** 10.3390/biology10070680

**Published:** 2021-07-18

**Authors:** Paulo Branco, Pedro Segurado, Maria João Costa, Afonso Teixeira, José Maria Santos, Maria Teresa Ferreira, Gonçalo Duarte

**Affiliations:** Forest Research Centre, School of Agriculture, University of Lisbon, Tapada da Ajuda, 1349-017 Lisboa, Portugal; psegurado@isa.ulisboa.pt (P.S.); mjcscosta@hotmail.com (M.J.C.); afonsolest@gmail.com (A.T.); jmsantos@isa.ulisboa.pt (J.M.S.); terferreira@isa.ulisboa.pt (M.T.F.); goncalofduarte@isa.ulisboa.pt (G.D.)

**Keywords:** fish conservation, endangered fish, expert judgement, species conservation status, species threats

## Abstract

**Simple Summary:**

This study aims to understand if the threats to freshwater-dependent species identified by The International Union for Conservation of Nature Red List of Threatened Species are correctly supported by valid literature. The results show that 99% of threats are not supported by validated published scientific knowledge. This may lead to ineffective conservation and management plans. Funding to study and fill baseline knowledge gaps about threats should be a priority.

**Abstract:**

Freshwater ecosystems are disproportionally important for biodiversity conservation, as they support more than 9% of known animal species while representing less than 1% of the Earth’s surface. However, the vast majority of the threats (99%, or 826 out of 837) identified by the International Union for Conservation of Nature Red List of Threatened Species known to affect the 434 known freshwater-dependent fish and lampreys of Europe are not supported by validated published scientific knowledge. This general lack of information about freshwater-dependent fish and lamprey species may have deleterious effects on species conservation, and additional funding is required to fill baseline knowledge gaps.

## 1. Introduction

Freshwater ecosystems are disproportionally important for biodiversity maintenance [1,2] as they support 9.5% of all animal species [3] while covering less than 1% of the Earth’s surface and representing only 0.01% of the water volume [4]. In addition to being ubiquitous, rivers are intrinsically linked to human society’s development and, with it, the continuous, and ever-growing, exploitation of natural resources. The resulting loss of resources has led freshwater ecosystems to be one of the most threatened ecosystems in the world [4,5,6] and to the endangerment of several river-dependent species [2,7]. Freshwater-dependent fish species are particularly affected by pressures such as overexploitation, water pollution, flow modification, habitat destruction or degradation, changing climates, e-commerce, invasions by exotic species, infectious diseases, harmful algal blooms, expanding hydropower, longitudinal connectivity fragmentation, water abstraction, acidification, eutrophication, draining of wetlands, water warming, channelization, urbanization, emerging contaminants, engineered nanomaterials, microplastic pollution, light and noise interferences, declining calcium and freshwater salinisation [4,8]. Most species are secluded in a given river basin without the option to naturally relocate to another river catchment. Due to the particular hierarchical dendritic nature of river networks [9], all segments are affected by pressures originating in the upstream drainage area, thus resulting in cumulative pressures in the downstream direction. These threats to fish survival can be independent or can interact when they co-occur [6]. The correct identification of the threats, or their combination, affecting species is determinant for resource allocation towards species conservation and management [10,11]. This identification has traditionally suffered from the difficulty in establishing causality, especially for threatened or rare species for which experimental work or multi-population comparisons are difficult [12].

The International Union for Conservation of Nature (IUCN) Red List of Threatened Species (IUCN Red List Committee 2013) is probably the most widespread endeavour to identify the threats affecting species [13,14,15]. Determining the threat status of species for the Red List assessment is mostly based on the species population trend (abundance and distribution), and distribution range and range shifts. Threat identification is part of the process and is used to justify status definition, management options and risk forecast [16]. They are arguably the most relevant information for effective conservation actions [17]. These species and threat assessments are mostly supported by experts willing to contribute to species conservation, and they frequently rely on empirical knowledge and tend to be precautionary [17], conservation-wise. In the most scientifically prolific era ever, traditional and specialist knowledge, although extremely relevant, should, when possible, also be supported by available knowledge published in scientific literature. Some works analyse the nature of threats from existing data, showing that this is possible (e.g., [12]).

The lack of support from valid literature that identifies threats has three probable causes that may not be mutually exclusive: (1) scientific knowledge gaps; (2) limited time-frame given to species evaluators to explore the published literature that may at times be overwhelming; and (3) use of a suboptimal process for identifying and reporting threats under the Red List species assessment. This work aims to understand if the threats to freshwater-dependent species identified by the IUCN during their species assessments are correctly supported by valid literature and to highlight potential consequences thereof.

## 2. Materials and Methods

To understand the validity of the threats identified by experts as affecting native freshwater fish species and lampreys of Europe, we identified species migratory phenology and conservation statuses, compiled all of the threats identified and their respective categories, and recorded which were directly supported by a reference. Afterwards, all available references were assessed to determine if they supported the threat identified or were misused (all articles, books and reports were read by the same author to maintain the same validity evaluation standard); this process was conducted by assessing the information present in the document with the threat it was supporting, and the source was considered valid when the information was clearly related to that species and supported the threat the reference was describing. The information retained was based on the European assessment of the species retrieved from the IUCN Red List database (2020) [18]; thus, the threats were relative to the study area, with the exceptions of *Alosa pontica, Myoxocephalus quadricornis* and *Iberochondrostoma olisiponensis*, for which the statuses were determined only at the global scope (all data are freely available at https://www.iucnredlist.org/ (accessed on 7 June 2020 ), Supplementary Material Table S1).

## 3. Results

**Figure 1:**
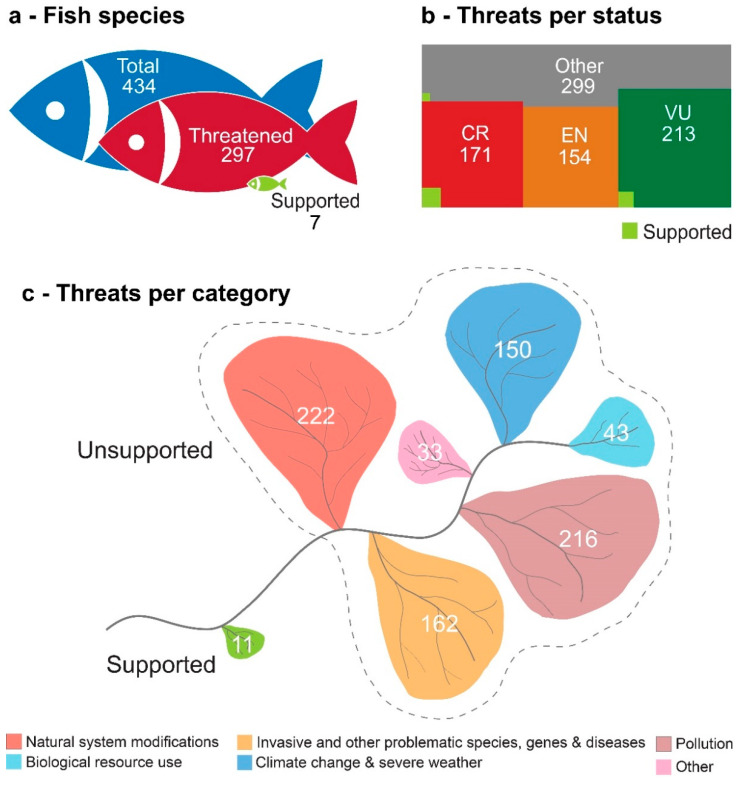
Isotype pictograms of (**a**) the number of fish and lamprey species (total, threatened and at least one threat supported by valid references—the literature is considered valid after assessing the information present in the document in relation to the threat referenced by it); (**b**) the number of threats attributed to each International Union for Conservation of Nature Red List species assessment (IUCN) species status (CR—Critically Endangered; EN—Endangered; VU—Vulnerable; Other—Remaining statuses; Supported—Number of threats supported by valid references); and (**c**) the number of threats by threat category according to the IUCN category list (Other—Threats belonging to the remaining IUCN threats categories; Supported—Number of threats supported by valid references).

A total of 434 species were included in the analysis, which resulted in 837 threats being identified for 297 species (137 species had no threats identified) (Table 1). Of the 837 threats identified, the majority (64%) were identified for species assigned a threat status (Critically Endangered, Endangered or Vulnerable) (Figure 1), highlighting the importance of threat identification for conservation efforts. There were 37 references, from 24 documents (58% of which were published scientific articles), supporting 27 threats affecting 13 different species. There were three documents not available online, which means they are inaccessible to most researchers and managers; these documents were referenced four times in the database and represented three otherwise-unsupported threats. Data resulting from these three references were excluded from the subsequent analysis. After reading the 21 available documents, 55% (18 out of 33) of the references adequately supported the threat they were referencing, meaning that only 46% (11 out of 24) of the threats with references were well supported and that only 58% (7 out of 12) of the species have correctly supported threats. Overall, only ca. 1% of the threats identified were supported by valid literature references, and less than 2% of the evaluated species have threats correctly supported by the literature.

## 4. Discussion

The database shows that not only are a very limited number of threats and their connection to species supported by the literature but also, more importantly, almost half of the references used do not support the threat for which they are used. This represents a twofold problem, as there is possibly little valid published information available [19] and the information that exists is being improperly used or used in an overly speculative fashion, or the evaluators were not given enough time to assimilate the available published information. Although understandable in the context of the precautionary principle in biodiversity conservation [17], the identification of threats by excess can lead to increasing difficulties in species conservation, having possible negative feedback effects, as managers usually face budget constraints and need to prioritize resource allocation. This non-literature-supported conservative approach hinders managers from correctly identifying and prioritizing the most relevant threats due to an over-dispersion of threats to endangered species and thus detracts from ecosystem rehabilitation and species conservation, which is the very purpose of identifying threats under the species conservation status assessment. This threat-identification process can be affected by a lack of knowledge, which is understandable for newly discovered or described species, for which not enough time has elapsed for research to be funded, conducted and published. Another reason for knowledge gaps is the lack of funding [20] for baseline research on individual species and for the continuous data collection to acquire long-term time-series, which thus allows for a causality nexus determination between specific threats and trends in species abundance and distribution. There also may exist a prevalence of funding for well-known species or regions [21], leading to unbalanced data availability and weak scientific baseline information for some species or regions [22].

There is a slightly higher percentage of threats supported by valid literature on the threats identified as affecting anadromous fish when compared to threats affecting other species. This may be because, even though these species spend only part of their life in fresh water and only a few species are present in Europe, their present and historic socioeconomic importance generated a larger body of knowledge [23]. Due to this, diadromous species (e.g., salmon), which are economically relevant resources for human populations, have more complete records in the IUCN Red List database [15]. The lack of scientific literature support in threat identification is also related to the fact that species’ Red List threat assessment is heavily based on expert empirical knowledge and traditional or local knowledge. Although very relevant and important, this type of knowledge may carry a high degree of uncertainty due to the difficulty that may arise in assessing the reliability of the account. The integration of this wealth of empirical knowledge in the IUCN Red List is extremely important since it allows for a more comprehensive evaluation of the species status. Nonetheless, considering threat identification, there needs to be a more systematic way to identify when this empirical knowledge was used, the uncertainty associated with it and the spatial extent to which it is valid. A given species may be affected by a given threat in only a portion of their distribution range, even if the threat is present throughout the species distribution range. There may exist local environmental and habitational particularities that potentiate or ameliorate the potential effect of a given threat or threat combination. The traditional and scientific empirical and published knowledge may at times refer to a portion of the spatial distribution of species, and the information associated with threat identification should reflect this to allow managers to properly address species conservation. Nonetheless, any degree of dependence on non-traceable information for the identification of threats affecting freshwater-dependent fish species may result in problems for future Red List species threat identification; as with the increasing competitiveness in academia, researchers tend to widen their research focus to increase their scientific outputs, and true specialists for a given species, species group or geographic region are becoming scarce, as is the traditional use of natural resources and thus of its specific knowledge. Research is also becoming more directed to processes and functions, to wider geographic scales and to mechanistic and predictive empirical or process modelling. Additionally, management or conservation-oriented research is increasingly catered towards communities rather than to populations [24]. This shift in research goals, i.e., away from a species-based focus, is sometimes dictated by editorial decisions favouring wide-audience-reaching articles and affects the availability of baseline information for specific species and its taxonomy, thus further affecting a data-informed definition of present and future species-level threat identification.

The present article highlights that threat identification, on which most species conservation efforts are built, is frequently based on expert empirical knowledge and is generally unsupported by scientific literature. This means the bulk of the scientific knowledge employed for threat identification is mostly expert-based and is thus unavailable for validation or managers to bespoke conservation actions. There is an actual peril of losing species-specific fundamental research due to a lack of interest, opportunity or funding. This lack of information, plus unpublished and thus mostly unavailable knowledge, forces experts to be over-conservative in their identification of threats, an understandable but undesirable approach that can backfire by not allowing effective species-specific or regional-specific conservation and management planning. The IUCN Red List should be considered a warning signal to alert managers to species in trouble, and managers should strive to conduct rigorous surveys to better define their conservation practices.

Threat identification should shift towards a more informed and supported procedure [12,19,25], e.g., using monitoring databases (e.g., https://www.eea.europa.eu/ (accessed on 1 April 2021)), with a further effort to relate these with species and pressures (https://www.eea.europa.eu/data-and-maps/ (accessed on 1 April 2021)) and to improve the understanding of the causes of species decline and not only symptoms that determine conservation status definition sensu IUCN Red List [26]. This change would enhance the reliability of the Red List threat assessment, empowering overall species conservation and threat management. More funding should be directed towards species-specific research and the publication of localized knowledge. The IUCN Red List species assessment is an excellent tool for conservation. The fact that most of the species’ threats identified therein are backed by empirical, traditional or local knowledge enforces the call for funding and publication mediums of the local restricted knowledge made above. At this moment, the IUCN Red List acts as a medium for the integration of this knowledge, but its report is not systematic and, as such, it is not a proxy for actual data publication and may be lost knowledge in the future. By increasing data scrutiny and by introducing data description, authorship and traceability, IUCN Red List, especially with its recognized rigorous peer inspection and review, may also be a valid repository for verified species threat traditional knowledge. These actions, if implemented, may positively affect the conservation of species, an intangible natural value.

## Figures and Tables

**Table 1:** Number of identified threats, number of threats supported by valid references (the literature is considered valid after assessing the information present in the document in relation to the threat referenced by it) and % of threats that are supported by a valid reference, classified according to the International Union for Conservation of Nature Red List species assessment (IUCN) Status, Migratory phenology (Phenology) and Threat typology (Threat). Here, only typologies with references in the IUCN Red List site were analysed. Thus, the total was not the same. In this table, all references and literature elements were considered, and literature not available were considered non-valid references herein. Critically Endangered (CR), Endangered (EN), Vulnerable (VU), Near Threatened (NT), Least Concern (LC) and Data Deficient (DD).

IUCN Status	Threats	Threats Supported by Valid References	% of Threats with Valid References	Phenology	Threats	Threats Supported by Valid References	% of Threats with Valid References	Threat		Threats	Threats Supported by Valid References	% of Threats with Valid References
CR	171	6	3.5	Resident	641	7	1.1	Biological resource use	Fishing and Harvesting Aquatic Resources	42	1	2.4
EN	154	0	0.0	Potamodromous	107	0	0	Human intrusions and disturbance	Work and Other Activities	2	0	0
VU	213	4	1.9	Anadromous	83	4	4.8	Natural system modifications	Dams and Water Management/Use	222	1	0.5
NT	44	0	0.0	Catadromous	5	0	0	Invasive and other problematic species, genes and diseases	Invasive Non-Native/Alien Species/Diseases	146	0	0
									Problematic Native Species/Diseases	8	2	25
									Introduced Genetic Material	12	2	16.7
LC	210	1	0.5	Amphidromous	1	0	0	Pollution	Domestic and Urban Waste Water	58	1	1.7
									Industrial and Military Effluents	52	0	0
									Agricultural and Forestry Effluents	106	1	0.9
									Garbage and Solid Waste	2	1	50
DD	45	0	0.0	-	-	-	-	Climate change and severe weather	Habitat Shifting and Alteration	2	2	100
									Droughts	147	0	0
TOTAL	837	11	1.3	TOTAL	837	11	1.3	TOTAL		799	11	1.4 (799); 1.3 (837)

## Data Availability

All data are freely available at https://www.iucnredlist.org/ (accessed on 7 June 2020).

## Supplementary Materials

The following are available online at https://www.mdpi.com/article/10.3390/biology10070680/s1, Table S1—Bibliographic references stated in The International Union for Conservation of Nature (IUCN) Red List of Threatened Species (2020) as support for identified threats (represented levels 1 and 2 of threats according to IUCN definitions) to European native freshwater dependent species. Status acronyms: Critically Endangered (CR), Endangered (EN), Vulnerable (VU), Near Threatened (NT), Least Concern (LC), Data Deficient (DD).
